# Human mast cells exhibit an individualized pattern of antimicrobial responses

**DOI:** 10.1002/iid3.295

**Published:** 2020-03-28

**Authors:** Karen M. Garcia‐Rodriguez, Rajia Bahri, Clara Sattentau, Ian S. Roberts, Anu Goenka, Silvia Bulfone‐Paus

**Affiliations:** ^1^ Faculty of Biology, Medicine and Health, School of Biological Sciences, Lydia Becker Institute of Immunology and Inflammation University of Manchester Manchester UK; ^2^ Faculty of Science and Engineering, School of Materials University of Manchester Manchester UK; ^3^ Faculty of Life Sciences, School of Cellular and Molecular Medicine University of Bristol Bristol UK

**Keywords:** degranulation, *Escherichia coli*, *Listeria monocytogenes*, mast cell extracellular traps, mast cells, *Staphylococcus aureus*, *Streptococcus pneumoniae*

## Abstract

**Introduction:**

Mast cells (MCs) are tissue‐resident immune cells implicated in antibacterial responses. These include chemokine secretion, degranulation, and the release of mast cell‐extracellular traps, which are primarily dependent on reactive oxygen species (ROS) production. Our study investigated whether human mast cells (hMCs) develop individual response patterns to bacteria located at different tissue sites: *Escherichia coli* (gut commensal), *Listeria monocytogenes* (foodborne intracellular pathogen), *Staphylococcus aureus* (skin commensal and opportunistic pathogen), and *Streptococcus pneumoniae* (upper respiratory tract commensal and lung pathogen).

**Methods:**

After live bacteria exposure, hMCs were analyzed by a combined flow cytometry assay for degranulation, ROS production, DNA externalization, and for β‐hexosaminidase, chemokine, and prostaglandin release.

**Results:**

*L. monocytogenes* induced hMC degranulation, IL‐8 and MCP‐1 release coupled with DNA externalization in a novel hMC ROS independent manner. In contrast, *S. pneumoniae* caused ROS production without DNA release and degranulation. *E. coli* induced low levels of hMC degranulation combined with interleukin 8 and MCP‐1 secretion and in the absence of ROS and DNA externalization. Finally, *S. aureus* induced hMCs prostaglandin D2 release and DNA release selectively. Our findings demonstrate a novel hMC phenomenon of DNA externalization independent of ROS production. We also showed that ROS production, degranulation, DNA externalization, and mediator secretion occur as independent immune reactions in hMCs upon bacterial encounter and that hMCs contribute to bacterial clearance.

**Conclusions:**

Thus, hMCs exhibit a highly individualized pattern of immune response possibly to meet tissue requirements and regulate bacteria coexistence vs defense.

## Graphical abstract

In our study we have assessed human mast cell responses after their encounter with bacteria typically localized at tissue interfaces: *Escherichia coli* (gut commensal), *Listeria monocytogenes* (gut pathogen), *Staphylococcus aureus* (skin commensal), and *Streptococcus pneumoniae* (lung commensal). The human mast cell immune reactions included extracellular DNA secretion, reactive oxygen species (ROS) production and degranulation measured in a novel flow cytometric assay, as well cytokine/chemokine and prostaglandin D2 secretion. Our findings demonstrate that DNA externalization, ROS production, and mediator secretion occur as independent immune reactions in human mast cells upon bacterial encounter. Thus, these results suggest that human mast cells exhibit a highly individualized pattern of antimicrobial responses.

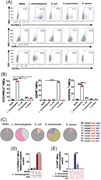

**Abbreviations**
*E. coli*: *Escherichia coli*
hMCs: human mast cells
*L. monocytogenes*: *Listeria monocytogenes*
MCs: mast cells
MCETs: mast cell extracellular traps
ROS: reactive oxygen species
*S. aureus*: *Staphylococcus aureus*
*S. pneumonia*: *Streptococcus pneumoniae*

## 1 INTRODUCTION

Mast cells (MCs) are immune cells that primarily reside at mucosal surfaces and the skin.^1^ MC progenitors either egress from the bone marrow and yolk sac to become mature MCs at tissue sites or differentiate locally under the influence of the tissue microenvironment.^2^, ^3^, ^4^ Recent studies have identified that MCs perform essential functions in response to pathogenic threats.^1^, ^5^, ^6^ MCs are strategically positioned to be among the first cells to interact with microbes, such as bacteria, and trigger specific immune responses against pathogens.^5^ Furthermore, the ability of MCs to rapidly release antimicrobial mediators and proinflammatory molecules has raised interest in the possibility of their therapeutic modulation for infectious diseases.^7^

MCs exhibit a range of defense mechanisms against bacteria that include the secretion of pro‐ and anti‐inflammatory mediators which shape both innate and adaptive immunity.^1^, ^5^ Such mediators can be released upon degranulation or following the engagement of the classical secretory pathway.^8^ For instance, *Mycobacterium tuberculosis* and *Enterococcus faecalis* are associated with MC cytokine release coupled with degranulation, whereas commensal bacteria including *Bifidobacterium bifidum*, do not induce MC degranulation.^9^, ^10^, ^11^ Mast cell extracellular trap (MCET) formation is a defense mechanism that exposes microbes to inflammatory mediators within a mesh of DNA.^12^, ^13^ MCETs have shown to contribute to antibacterial immunity, including infections caused by *Listeria monocytogenes*, *Streptococcus pyogenes*, and *E. faecalis*.^13^

Neutrophils form extracellular traps by two mechanisms.^14^, ^15^ The first involves cell death and occurs through decondensation of the nuclear envelope that is triggered by the presence of reactive oxygen species (ROS).^14^, ^16^, ^17^ The second involves DNA release without compromising cell viability and independently of ROS production.^18^ However, in MCs, only ROS‐dependent extracellular traps have been described^19^, ^20^ with the majority of studies performed in animal models or human cell lines.^19^ Furthermore, MCET formation seems to depend on specific bacterial stimulation. For instance, DNA release is observed after *L. monocytogenes* or *E. faecalis* encounter, whereas release of DNA is inhibited after *M. tuberculosis* exposure.^19^, ^21^

We hypothesized that different microbes induce individual patterns of functional response by MCs, including degranulation, chemokine secretion, and DNA release in the presence or absence of ROS production. We, therefore, assessed human mast cell (hMC) responses after their encounter with bacteria typically localized at tissue interfaces: *Escherichia coli* (gut commensal), *L. monocytogenes* (foodborne intracellular pathogen), *Staphylococcus aureus* (skin commensal and opportunistic pathogen), and *Streptococcus pneumoniae* (upper respiratory tract commensal and lung pathogen). We found that *L. monocytogenes* induced hMC degranulation and DNA release in the absence of ROS production while *S. pneumoniae* promoted ROS production without inducing DNA release and degranulation. Furthermore, *E. coli* promoted hMC degranulation in the absence of DNA or ROS release, whereas *S. aureus* did not induce hMCs to degranulate or release DNA but selectively induced MC prostaglandin D2 (PGD2) secretion. These data suggest that hMCs display specific patterns of response according to the individual pathogen, involving degranulation, DNA release, ROS production, and chemokine release. These activities occur as independent reactions. Therefore, our findings suggest that hMCs respond strategically to individual threats orchestrating unique immune responses in bacterial defense.

## 2 EXPERIMENTAL PROCEDURES

### 2.1 Bacterial strains

Bacterial strains were kept as stocks at −80°C in 15% glycerol. Before bacteria were used, growth curves were generated to correlate optical density (OD) with colony‐forming units (CFUs). *E. coli* (ATCC 25922) and *S. aureus* (ATCC 25923) were cultured in lysogeny broth (Sigma‐Aldrich, St Louis, MO) and *L. monocytogenes* (inlA) in tryptic soy broth (TSB) (Sigma‐Aldrich), at 37°C under shaking incubator (200 rpm). *S. pneumoniae* strain D39 (R6) was cultured in Todd Hewitt Broth and yeast extract (0.5%) at 37°C without shaking. Bacterial strains were cultured and harvested at a midpoint of the log‐growth phase (OD: 0.3‐0.6) for cell stimulations.

### 2.2 Human primary MC culture

hMCs were generated as previously described.^56^ Briefly, cells were obtained by a positive selection of CD117^+^ haematopoietic progenitors obtained from buffy coat blood mononuclear cells by immunomagnetic sorting (Miltenyi Biotec, Bergisch Gladbach, Germany). Cells were cultured for 4 weeks in Iscove's modifiied Dulbecco's medium with GlutaMAX‐I supplemented with 50 µmol/L β2‐mercaptoethanol, 0.5% bovine serum albumin (BSA), 1% insulin‐transferrin‐selenium, 100 U/mL penicillin, 100 µg/mL streptomycin (Invitrogen, Carlsbad, CA), ciprofloxacin (Bio‐world), human interleukin 6 (IL‐6) (50 ng/mL; PeproTech, Rocky Hill, NJ), human IL‐3 (10 ng/mL; PeproTech), human stem cell factor (SCF) (100 ng/mL; PeproTech), and StemRegenin (1 µM; Cayman). Cells were progressively transferred to culture media containing Iscove's modified Dulbecco's medium with GlutaMAX‐I supplemented with 50 µmol/L β2‐mercaptoethanol, 0.5% BSA, 1% insulin‐transferrin‐selenium, 100 U/mL penicillin, 100 µg/mL streptomycin, human IL‐6 (50 ng/mL), and human SCF (100 ng/mL) for 4 weeks. After 8 to 10 weeks of culture, cells were tested for purity and maturity, measuring CD117 and FcεRIa expression.

### 2.3 hMC infection

hMCs were washed three times to remove antibiotics and plated at a density of 5 × 10^5^ cells/mL in supplemented medium without antibiotics. Cells were rested at 37°C in 5% CO_2_ for 1 hour before infections. Bacteria were cultured and prepared at a multiplicity of infection (MOI) of 25:1 in supplemented medium without antibiotics. MOI (25:1) was selected by testing different bacterial concentrations and choosing the minimum MOI able to induce hMC degranulation. Each bacterial species was incubated with plated hMCs for 2 hours at 37°C, 5% CO_2_. Supplemented media without antibiotics was used to incubate unstimulated controls.

### 2.4 Flow cytometry

For the measurement of ROS, hMCs were pretreated with dihydrorhodamine 123 (DHR 123) (Thermo Fisher Scientific) at 1.33 µg/mL for 15 minutes before stimulation. To inhibit ROS, 10 µM diphenyleneiodonium (DPI) (Sigma‐Aldrich) was added 30 minutes before stimulation. Once stimulated with the four different bacteria, hMCs were washed several times with phosphate‐buffered saline (PBS) before staining. Cells were incubated with Fc‐block and stained with fixable Live/Dead Zombie NIR (BioLegend) according to the manufacturer's protocol to exclude dead cells with compromised cell membranes.

Cells were stained with anti‐human antibodies CD107a (LAMP‐1) (H4A3; BD BioLegend) and analyzed by flow cytometry as above. Degranulation was measured as a percentage of CD107a^+^ cells.

TO‐PRO‐3 (Invitrogen) was added to cells in the final 15 minutes of staining at 1 µM. All dyes and antibodies were diluted in flow cytometry staining buffer and incubated for 30 minutes at 4°C before washing and fixing cells with 4% paraformaldehyde. Flow cytometric data were acquired using the BD LSR‐II. Single stain controls were prepared using compensation beads (OneComp eBeads; Thermo Fisher Scientific). The postacquisition data analysis was performed using FlowJo software (Treestar version 10.4.2).

### 2.5 Confocal microscopy

hMCs were washed several times to remove antibiotics and seeded on a glass slide precoated with poly‐d‐lysine at a concentration of 5 × 10^4^ hMCs/mL. Cells were left untreated (control) or incubated with *L. monocytogenes* for 16 hours at a MOI 25:1. After stimulation, cells were washed three times and fixed with formaldehyde 4% for 20 minutes and permeabilized with Triton (0.2%) (Sigma‐Aldrich). After three washes cells were incubated first with blocking buffer (10% goat serum in PBS) for 1 hour and then with mouse anti‐human tryptase antibody (ab2378; Abcam) for 1 hour followed by 1 hour incubation with goat anti‐mouse antibody conjugated to Alexa 647 fluorophore (ab150115; Abcam). Cells were washed three times and mounted with fluoroshield mounting media with 4′,6‐diamidino‐2‐phenylindole (DAPI; Abcam) to be analyzed by confocal microscopy. Images were collected at a zoom factor of 2.58 using a Leica TCS SP8X inverted confocal microscope equipped with a tuneable white light laser (WLL), and a diode 405 nm laser, a ×40/0.85 dry objective, and HyD hybrid detectors. AL647 fluorescence was excited at 647 nm with WLL laser and detected at 655 to 718 nm on a HyD point detector. DAPI fluorescence was excited at 405 nm using the UV laser and collected pn a HyD detector at 410 to 470 nm. Confocal three‐dimensional stacks were acquired with a depth of 8 µm using Leica LASX software. Images stacks were then processed, sum projected and analyzed using Fiji.^57^

### 2.6 β‐Hexosaminidase assay

hMCs were stimulated with *L. monocytogenes* (100 µL, 5 × 10^5^ cells/mL) for 2 hours. After incubation, supernatants were harvested and cell pellets were lysed in 1% Triton X‐100. β‐Hexosaminidase activity was measured in supernatants as well as in cell pellets by adding the substrate *p*‐nitrophenyl *N*‐acetyl‐β‐d‐glucosamine at 1 mmol/L (Sigma‐Aldrich) in 0.05 mol/L citrate buffer (pH 4.5) for 2 hours at 37°C in a 5% CO_2_ atmosphere.^56^ The reaction was stopped by using 0.05 mol/L sodium carbonate buffer (pH 10). OD was measured at 405 nm. Degranulation was assessed as the percentage release of total β‐hexosaminidase.

### 2.7 Measurement of IL‐8, MCP‐1, and PGD2

After the incubation of hMCs with the different bacterial strains, cells were centrifuged, and supernatants were collected to measure mediator content. Supernatants were either used immediately after collection or frozen at −80°C before being analyzed. PGD2 levels were measured according to the manufacturer's protocol using enzyme‐linked immunosorbent assay chemical Cayman Kit (cat 412012). IL‐8 and MCP‐1 were quantified by a BD cytometric bead array multiplex kit following manufacturer's protocol using a FACSVerse flow cytometer. The analysis was performed using the FCAP Array Software v3.0.

### 2.8 CFU assay

hMCs were stimulated for 2 hours with *L. monocytogenes* at a MOI 25:1 in 50 µL of cell media. Fifty microlitres of initial stocks of *L. monocytogenes* (corresponding to MOI of 25:1) were incubated alone for 2 hours. After incubation, 100 µL of cold‐sterile ultrapure water was added on the top of cell‐bacterial suspensions to lyse hMCs, and incubated for 10 minutes at 4°C. Then, serial dilutions 1:10 to 1:10^8^ were prepared in PBS, and 10 µL of each dilution was plated on TSB agar (Sigma‐Aldrich) in five replicates (50 µL in total). CFUs were counted after 1 to 3 days (3 days final count).

### 2.9 Statistical analysis

Data were analyzed using the GraphPad Prism 7.0 (GraphPad Software, San Diego, CA). All samples were processed in triplicates. Comparison between more than two groups was achieved using one‐way analysis of variance correcting for Tukey's multiple comparisons and comparisons between two groups were performed by unpaired *t* test. Correlations were calculated using Spearman *R* (*R* ^2^). *P* values of .05 or less were considered to be statistically significant.

## 3 RESULTS

### 3.1 *L. monocytogenes* and *E. coli* induce hMC degranulation

**Figure 1:**
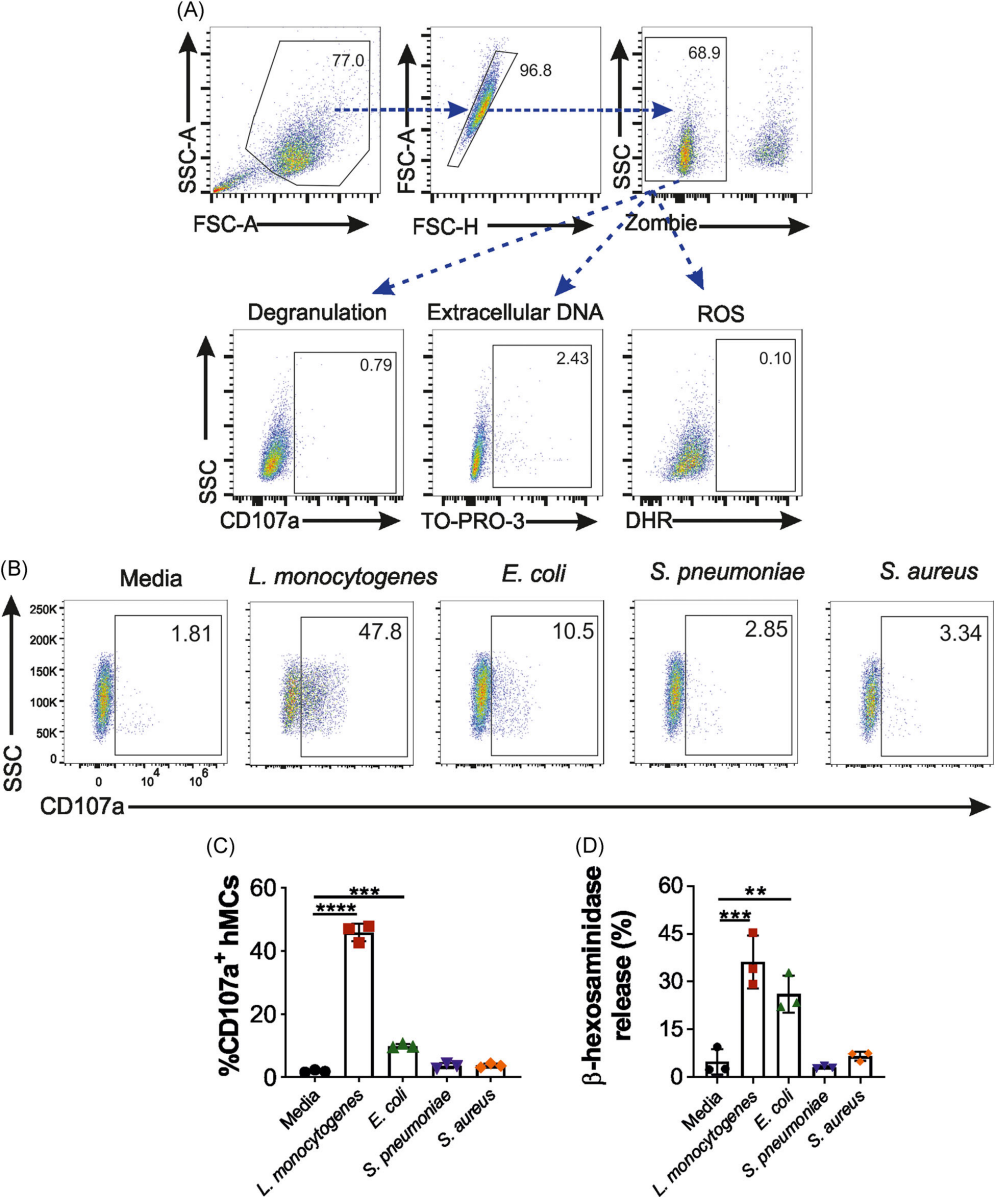
hMCs show different patterns of degranulation in response to different bacterial strains. hMCs were stimulated with either *Listeria monocytogenes* (*L. monocytogenes*), *Escherichia coli* (*E. coli*), *Streptococcus pneumoniae* (*S. pneumoniae*), or *Staphylococcus aureus* (*S. aureus*) at a MOI of 25:1 or media for 2 hours. A, Single live cells were selected by the side scatter (SSC‐A), forward scatter (FSC‐A). Live vs dead cells were discriminated by Zombie NIR staining. CD107a antibody and TO‐PRO‐3 and DHR dyes were used to investigate degranulation, DNA secretion and ROS production, respectively. B, CD107a flow cytometry representative of four independent experiments shows MC degranulation upon bacterial stimulation. C, Percentage of CD107a expressing cells (D) and % of β‐hexosaminidase release upon bacterial stimulation. Graphs (C) and (D) are a representative of four independent experiments performed each with three replicates. Statistical analysis was performed using two‐way ANOVA and Tukey's multiple comparisons test (****P < .0001, ***P < .001, **P < .01). ANOVA, analysis of variance; DHR, dihydrorhodamine; hMC, human mast cell; MC, mast cell; MOI, multiplicity of infection; ROS, reactive oxygen species

Several bacteria are known to trigger MC degranulation, whereas nonpathogenic gut resident strains, including *Lactobacillus* spp. appear to inhibit this process.^22^ We sought to characterize further and compare MC degranulation as a defense mechanism against bacteria encountered at different tissue sites. hMCs derived from peripheral blood precursors that had the phenotypic and functional properties of mature hMCs (Figures 1A and SA) were stimulated with an intracellular intestinal pathogen *L. monocytogenes*, an intestinal commensal *E. coli*, a skin commensal *S. aureus*, or a lung commensal *S. pneumoniae* intending to detect differences in induced hMC degranulation.

To assess hMC degranulation following incubation with bacteria at a MOI of 25:1, we measured: (a) surface expression of CD107a (Figure 1B,C)^23^ using flow cytometry; and (b) granule compound release by means of β‐hexosaminidase secretion (Figure 1D). Unstimulated hMCs were used as a negative control.

The level of degranulation (CD107a^+^ hMCs; β‐hexosaminidase release) was highest in hMCs incubated with *L. monocytogenes* (45.8%; 36.1%), compared with *E. coli* (9.8%; 26.1%), *S. pneumoniae* (3.7%; 2.9%), and *S. aureus* (3.7%; 6.4%) exposure (Figure 1B‐D). Both *S. aureus* and *S. pneumoniae* showed no significant effect on hMC degranulation (1.9%) (Figure 1B‐D).

Furthermore, the percentage of β‐hexosaminidase release was found to correlate (*r*_s_ = 0.82) with the CD107a hMC expression (Figure SB). Thus, hMCs exhibit a diverse degranulation response to different bacterial strains, and obligate pathogens invading gut, such as *L. monocytogenes*, may promote higher levels of MCs degranulation compared with opportunistic pathogens resident in skin or lung.

### 3.2 Selective bacterial stimulation induces hMCs to release IL‐8, MCP‐1, and PGD2

Among the studied bacteria, only *L. monocytogenes* and *E. coli* induced hMC degranulation. However, hMCs also release a wide variety of proinflammatory mediators independently of degranulation in a stimuli‐specific manner.^8^ Such proinflammatory molecules allow hMCs to orchestrate the response of other immune cells to bacteria.^24^ We hypothesized that bacteria encountered at different tissue sites induce a specific pattern of cytokine production independently of degranulation. To test this hypothesis, hMC mediators (MCP‐1, IL‐8, and PGD2) were quantified in culture supernatants after stimulation with bacteria.

**Figure 2:**
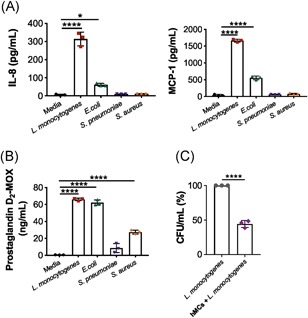
Bacterial stimulation induces selective IL‐8, MCP‐1, and prostaglandin D2 secretion and hMC‐mediated bacterial clearance. hMCs were stimulated with either *Listeria monocytogenes* (*L. monocytogenes)*, *Escherichia coli* (*E. coli*), *Streptococcus pneumoniae* (*S. pneumoniae*), or *Staphylococcus aureus* (*S. aureus*) at a MOI of 25:1 or media control for 2 hours. Supernatants were collected for measurement of (A) IL‐8 and MCP‐1 and (B) prostaglandin D2‐MOX concentrations by a multiplex cytometric bead array. C, To analyze bacterial survival, hMCs were stimulated with *L. monocytogenes* at a MOI of 25:1 for 2 hours. Bacterial inoculums were used as control bacterial concentration (100%) and treated equal to stimulated hMCs. After incubation, samples were lysed, serial dilutions were prepared, cultured in agar plates, and CFUs were counted. Data shown are the mean of three replicates of a representative experiment out of four independent experiments performed. Statistical analysis was performed using unpaired *t* test (C) and two‐way ANOVA and Tukey's multiple comparisons test (A,B) (****P < .0001, ***P < .001, **P < .01, *P < .1). ANOVA, analysis of variance; CFU, colony‐forming unit; hMC, human mast cell; IL‐8, interleukin 8; MOI, multiplicity of infection

*L. monocytogenes*, which induced the strongest degranulation among the studied bacteria (Figure 1B‐D) significantly promoted the release of proinflammatory mediators (mean chemokine release) including IL‐8 (314.2 pg/mL) and MCP‐1 (1663 pg/mL) secretion compared with unstimulated cells (Figure 2A). *E. coli*, induced a relatively low degranulation (Figure 1B‐D) and IL‐8 (61.2 pg/mL) and MCP‐1 (553.9 pg/mL) secretion (Figure 2A) compared with *L. monocytogenes*. The levels of hMC mediator secretion upon *S. aureus* and *S. pneumoniae* exposure were similar to the unstimulated controls. The release of granulocyte‐macrophage colony‐stimulating factor, IL‐10 and IL‐1β was tested but was undetectable (data not shown).

*L. monocytogenes*, *S. aureus*, and *E. coli* significantly induced PGD2 secretion with mean levels of 65.9, 27.4, and 62.1 ng/mL, respectively. No significant PGD2 release compared with unstimulated controls (0.6 ng/mL) was observed upon *S. pneumoniae* stimulation (9 ng/mL) (Figure 2B). Taken together, our findings suggest that mediator release is both bacterial specific and independent of degranulation.

### 3.3 hMCs contribute to *L. monocytogenes* killing

hMCs produce high levels of extracellular DNA, IL‐8, MCP‐1, and PGD2 upon *L. monocytogenes* stimulation. Different studies have shown that cytoplasmic content exposed in DNA traps is able to kill pathogens.^12^, ^13^, ^25^ Therefore, we aimed to investigate whether the hMC mediator secretion induced by *L. monocytogenes* infection affects bacterial survival. After hMCs incubation with *L. monocytogenes*, bacterial numbers were determined by CFUs. As observed in Figure 2C, 55% less CFUs were observed in the cultures containing hMCs compared with the ones of bacteria alone. These findings suggest that hMC responses to *L. monocytogenes* contribute to bacterial clearance.

### 3.4 hMCs display a distinctive pattern of degranulation, DNA secretion, and ROS production upon bacterial encounter

hMCs contribute to bacterial containment through the formation of extracellular traps. Current literature reports MCET formation upon MC exposure to *L. monocytogenes*, *S. aureus*, *S. pyogenes*, and *M. tuberculosis*.^19^, ^20^ However, DNA secretion, reflective of MCET formation, has been reported as dependent on ROS production. We have shown that the complex nature of hMC responses allows degranulation and mediator secretion to be discrete and independent processes. Thus, we hypothesized that bacterial‐induced DNA release might occur in a bacterial specific and ROS independent manner, as in neutrophils.^18^ To investigate this idea, hMCs were incubated with *L. monocytogenes*, *E. coli*, *S. pneumoniae*, or *S. aureus* for 2 hours and stained with CD107a antibodies, TO‐PRO‐3 and DHR dyes to assess degranulation, DNA release, and ROS production, respectively.

**Figure 3:**
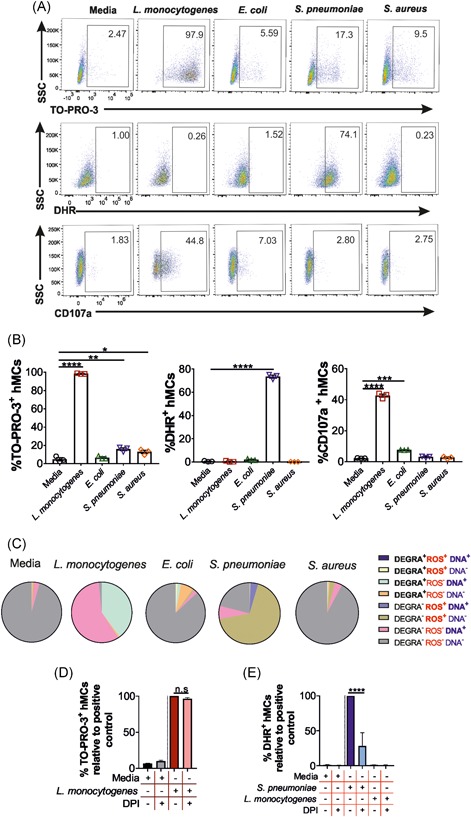
The MC pattern of degranulation, DNA secretion, and ROS production is bacteria specific. hMCs were stimulated with either *Listeria monocytogenes* (*L. monocytogenes*), *Escherichia coli* (*E. coli*), *Streptococcus pneumoniae* (*S. pneumoniae*), or *Staphylococcus aureus* (*S. aureus*) at a MOI of 25:1 or media controls for 2 hours. After stimulation, cells were stained with TO‐PRO‐3 (DNA, DNA secretion), DHR (ROS, ROS production), and CD107a (DEGRA, degranulation) and analyzed by flow cytometry. A, Flow cytometry data show one representative plot. B, Percentage of TO‐PRO‐3^+^, DHR^+^, and CD107a^+^ cells after bacterial stimulations. Each graph shows the mean of three replicates of a representative experiment out of four independent experiments performed. Statistical analysis was performed using two‐way ANOVA and Tukey's multiple comparisons tests (****P < .0001, ***P < .001, **P < .01, *P < .1). C, Frequency of CD107a^+/−^ (DEGRA, black), DHR^+/−^ (ROS, red) and TO‐PRO‐3^+/−^ (DNA, blue) cells upon bacterial exposure. Data for each condition (*L. monocytogenes*, *E. coli*, *S. pneumoniae*, *S. aureus*, and media control) was taken from the mean (n = 3) of a representative experiment of four independent experiments. To investigate DNA release is ROS independent, hMCs were incubated with DPI to inhibit ROS production, and stimulated then with *L. monocytogenes* and stained with TO‐PRO‐3. A, Values were normalized to the TO‐PRO‐3 positive control hMCs challenged with *L. monocytogenes* in the absence of DPI. B, The inhibitory effect of DPI on ROS production was controlled in hMCs stimulated with *S. pneumonia*, and stained with DHR. Data show values normalized to hMC incubated with *S. pneumonia* without DPI. ANOVA, analysis of variance; DHR, dihydrorhodamine; DPI, diphenyleneiodonium; hMC, human mast cell; MC, mast cell, MOI, multiplicity of infection; ROS, reactive oxygen species

Different levels of DNA externalization (mean %TO‐PRO‐3^+^ cells), ROS release (mean %DHR^+^ cells), and degranulation (mean %CD107a^+^ cells) were observed among stimulated cells (Figure 3A‐C). *L. monocytogenes* induced in hMCs a significantly higher DNA externalization (98.4%) compared with unstimulated controls (4.9%; Figure 3B). *L. monocytogenes*‐dependent degranulation and DNA secretion occurred in the absence of ROS production (Figure 3A,B). *E. coli* induced lower degranulation compared to *L. monocytogenes* (Figure 1) occurring in the absence of DNA secretion and ROS production (Figure 3A,B). In contrast, ROS production upon *S. pneumoniae* stimulation (73.5%) was significantly higher compared with unstimulated controls (0.6%). Interestingly, despite the high numbers of hMCs producing ROS after *S. pneumoniae* stimulation, only 16% of hMCs released DNA and in the absence of degranulation (Figure 3A,B). In addition, *S. aureus* significantly induced DNA externalization (12.9%) in hMCs compared with unstimulated controls occurring in the absence of ROS production and degranulation (Figure 3A,B).

To investigate the association of hMC degranulation with DNA secretion and ROS production after exposure to the four studied bacteria, we carried out a part of a whole analysis correlating degranulation (CD107a^+^), ROS production (DHR^+^), and extracellular DNA release (TO‐PRO‐3^+^) in stimulated hMCs (Figure 3C). Upon *L. monocytogenes* exposure, 40% of hMCs were CD107a^+^, DHR^−^, and TO‐PRO‐3^+^ (Figure 3C, green) and 60% were CD107a^−^, DHR^−^, and TO‐PRO‐3^+^ (Figure 3C, pink). This indicates that although most of the *L. monocytogenes*‐stimulated cells secreted DNA (98%), only 60% were associated with degranulation and none of the total population produced ROS. In contrast, degranulated cells stimulated by *E. coli* were not associated with DNA and ROS production. Furthermore, after *S. pneumoniae* encounter, 67% of cells produced ROS in the absence of DNA and degranulation. In addition, only 4% of the total DNA released (17%) after *S. pneumoniae* exposure was associated with ROS. Finally, after *S. aureus* stimulation, DNA release and degranulation were not found to be linked.

To further prove that DNA release can occur in hMCs independently from ROS secretion, hMCs exposure to *L. monocytogenes* was preceded by incubation with DPI, a ROS inhibitor.^26^ TO‐PRO‐3 and DHR were measured by flow cytometry (Figure 3D,E). hMCs infection with *S. pneumoniae* was used as a positive control for DPI‐induced ROS inhibition (Figure 3E). As shown in Figure 3D, ROS inhibition did not affect DNA release.

Altogether, these findings suggest that in hMCs the release of DNA, ROS production, and degranulation can occur independently and under the control of distinct bacterial‐cell interaction.

### 3.5 *L. monocytogenes* infection affects hMC viability

**Figure 4:**
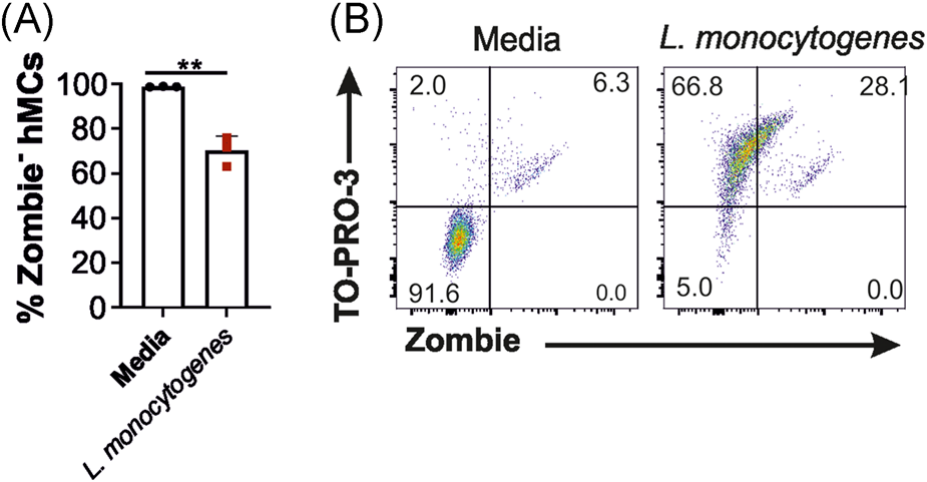
*L. monocytogenes* affects human mast cell (hMC) viability. hMC viability upon bacterial infection was measured after *L. monocytogenes* stimulation, cells were stained with (A) Zombie NIR (live cells) and (B) TO‐PRO‐3 (DNA releasing cells). Flow cytometry plots show one replicate of a representative experiment out of three independent experiments. Graph shows the mean of three replicates of a representative experiment out of three independent experiments performed. Statistical analysis was performed using unpaired *t* test (**P < .01)

We showed that *L. monocytogenes* induces an extensive release of DNA (assessed by TO‐PRO‐3) from hMCs (Figure 3). To investigate whether bacterial infection affects cell viability, hMCs were incubated with the four bacterial strains and cell viability (mean Zombie^−^ cells) was assessed by flow cytometry. While *E. coli*, *S. pneumoniae*, and *S. aureus*, did not affect hMC survival, *L. monocytogenes* reduced cell viability (Zombie^−^ cells, 70.2%) (Figures 4A and SC). Furthermore, most of the live cells (Zombie^−^) were TO‐PRO‐3^+^ (66.8%). However, we observed that 28% of hMCs releasing DNA (TO‐PRO‐3^+^) were positive for the cell death marker (Zombie^+^) (Figure 4B). Thus, these findings indicate that DNA release induced by *L. monocytogenes* occurs in live hMCs, and that this phenomenon is associated with a low level of cell death.

### 3.6 Infection of hMCs with *L. monocytogenes* induces the formation of extracellular traps

**Figure 5:**
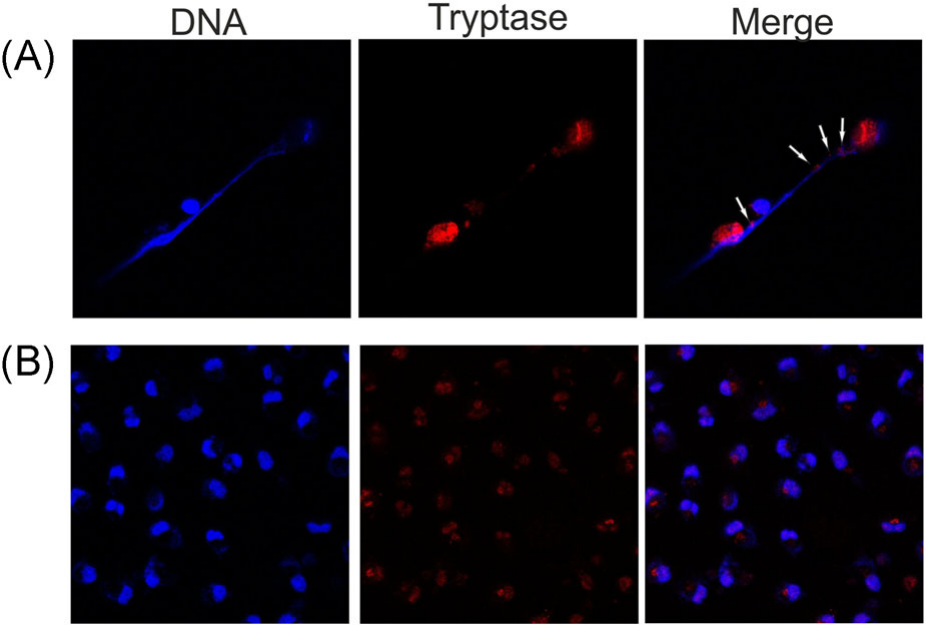
*L. monocytogenes* induces extracellular trap formation. Human mast cells were seeded in a glass slide precoated with poly‐d‐lysine. Cells were settled and stimulated with *L. monocytogenes* or media (unstimulated control) for 16 hours. After incubation cells were washed and stained for extracellular DNA using 4′,6‐diamidino‐2‐phenylindole and for tryptase with anti‐human tryptase antibody conjugated to Alexa 647 fluorophore. Slides were analyzed by confocal microscopy. A, *L. monocytogenes*‐stimulated cells and (B) unstimulated controls were stained and merged to visualize extracellular and intracellular tryptase‐DNA colocalization, respectively

MC lines and rodent MCs have been shown to release DNA using microscopy techniques.^19^, ^20^, ^21^, ^27^ To investigate whether the release of extracellular DNA in hMCs was associated with the formation of extracellular traps, cells were incubated with *L. monocytogenes* for 16 hours and colocalization of extracellular DNA (DAPI staining) and MC granule content release (tryptase staining) was analyzed by confocal microscopy. As shown in Figure 5, *L. monocytogenes*‐stimulated hMCs showed externalized DNA (Figure 5A) that colocalized with tryptase compared with unstimulated controls (Figure 5B). These data indicate that hMCs exposed to *L. monocytogenes* form extracellular traps.

## 4 DISCUSSION

The present study demonstrates that hMCs employ distinctive patterns of responses to *L. monocytogenes*, *E. coli*, *S. pneumoniae*, and *S. aureus*. The hMC immune reactions include DNA externalization, ROS production, degranulation and chemokine, and PGD2 secretion.

Differences between hMCs and rodent MCs or MC lines have been described.^28^ Furthermore, since hMCs are tissue‐resident cells, their location and relative low numbers makes it difficult to isolate and use them for complex experimental settings. Therefore, blood‐differentiated mature hMCs were used in this study as an alternative useful tool to tissue cells.

The release of extracellular traps is well characterized in neutrophils as a mesh of histones, antimicrobial peptides and granular proteins to trap and kill bacteria.^14^ Using a flow cytometry assay, we have found that *L. monocytogenes* and *S. aureus* induce DNA externalization without intracellular ROS production. This mechanism is novel in hMCs, which have so far been described to form extracellular traps (ETs) while undergoing NADPH‐dependent cell death.^19^, ^20^, ^21^ In neutrophils, the latter type of ET production is termed “suicidal NETosis” as it leads to cell death.^14^ However, in response to *S. aureus* Pilsczek et al^18^ described a second ROS independent mechanism in neutrophils called “vital NETosis” in which the release of DNA occurs by fusion of DNA‐containing vesicles with the plasma membrane.^18^ A similar mechanism of “vital MCETosis” might occur in ROS‐negative, DNA positive hMCs exposed to *L. monocytogenes*, *S. aureus*, and *E. coli* that would be in line with the long‐lived, tissue‐resident nature of hMCs.^29^ Our study demonstrated that *L. monoctyogenes* induces MCETs and a low level of cell death. Therefore, we suggest that, upon *L. monoctyogenes* encounter, hMCs exhibit early “suicidal MCETosis” as well as early “vital‐DNA release,” and following this, late (after 16 hours) DNA externalization and web‐like structures formation, late suicidal MCETosis. A similar process was described in neutrophils,^18^ where a ROS independent vital‐NETosis induces rapid DNA release (5‐60 minutes) without compromising the cell membrane.^18^ In MCs, the rapid release of DNA may contribute for the release of inflammatory mediators and bacterial clearance.

The pore‐forming toxin, listeriolysin O, is a primary virulence factor of *L. monocytogenes* that conveys phagocytosis avoidance and allows bacteria to reside intracellularly.^30^ Previous studies have shown that this toxin is necessary to trigger hMCs responses such as degranulation and mediator secretion.^31^ Although this needs to be confirmed, this toxin in addition to inducing tryptase secretion may be involved in the process of DNA release and MCET formation. In our study, *L. monocytogenes*, the only obligate pathogen tested, which also resides intracellularly, induced a robust MC response with active DNA release. In line with the extent of DNA release measured in our assay, Campillo‐Navarro et al^19^ observed a peak of DNA release in a hMC line after 3 hours exposure to *L. monocytogenes* by fluorescent microscopy. Primary hMCs generally respond faster to external triggers.^32^, ^33^ However, by microscopy, we demonstrated that *L*. monocytogenes induced DNA externalization colocalized with granular content (tryptase) only after 16 hours incubation. In contrast, flow cytometry demonstrated an early DNA externalization after only 2 hours incubation. Thus flow cytometry may provide a sensitive and more suitable detection technique for quantifying DNA release at early stages upon infection.

The rapid and robust release of DNA observed may serve MCs, which display low phagocytic capacity,^34^, ^35^ the ability to inhibit bacterial multiplication alongside MC degranulation together with chemokine and cytokine secretion which is vital in driving neutrophil recruitment during *L. monocytogenes* infection, as suggested by Gekara and Weiss.^36^ In our study, we confirmed that *L. monocytogenes* is killed by hMCs, while promoting hMC degranulation and the release of IL‐8 and MCP‐1, thus supporting the role of MC in the inflammatory process of cellular recruitment and bacterial clearance.

In previous studies, minimal degranulation was observed after exposing murine bone marrow–derived mast cells for 2 hours to *L. monocytogenes* (MOI: 10:1). Furthermore, a peak of MCP‐1 secretion was shown at 2 hours using a MOI of 100:1.^31^ In contrast, our findings showed a stronger hMC degranulation after *L. monocytogenes* exposure and similar concentrations of secreted MCP‐1 at a MOI of 25:1. This suggests that hMCs are more sensitive to pathogenic threats compared with mouse MCs.

MCs appear to exhibit a different range of functional responses toward commensal bacteria. Magerl et al^22^ demonstrated in vitro that high doses (1 × 10^9^ CFU/mL) of a probiotic *E. coli* strain could inhibit mouse MC degranulation. Furthermore, gut resident probiotic bacterial strains such as *Lactobacillus rhamnosus* and *Bifidobacterium animalis* downregulate degranulation‐inducing receptors.^37^ While degranulation may be redundant in the MC response to *E. coli*, the release of proinflammatory mediators, including tumor necrosis factor (TNF), IL‐8,^38^, ^39^, ^40^ and leukotrienes appear pivotal for *E. coli* infection recovery.^41^ In our study, *E. coli* strain ATCC 25922, originally a clinical strain was not associated with an inhibitory effect on degranulation, perhaps reflecting differences in the *E. coli* strains used by ourselves and Magerl.^22^ However, it induced a lower level of degranulation compared with *L. monocytogenes*. IL‐8 and MCP‐1 (CCL2) and PGD2 are important chemoattractants that recruit inflammatory cells in defense to pathogens.^42^ We showed the release of IL‐8, MCP‐1, and PGD2. This suggests hMCs may strategically contribute to cell recruitment during an acute infection against pathogenic bacteria by the selective release of chemokines while avoiding an enhanced inflammatory reaction caused by uncontrolled hMC degranulation upon nonpathogenic bacterial encounter. Furthermore, *E. coli* virulence factors activate MC responses. These include the type 1 fimbriae, which induces the release of TNF, IL‐6, and eicosanoids,^43^ and the pore‐forming toxin α‐hemolysin, which has been described to contribute to MC degranulation and IL‐8 release.^38^ Thus, these virulence factors may be involved in the chemokine secretion, degranulation, and PGD2 release observed in our hMC cultures.

*S. pneumoniae* is a Gram‐positive coccus that resides within the upper respiratory tract but can cause invasive sinopulmonary infection by its outgrowth.^44^ Pneumolysin is a crucial virulent factor of *S. pneumoniae*.^45^ Cruse et al^46^ observed that pneumolysin together with H_2_O_2_ are virulent factors causing hMC cytotoxicity. Our data demonstrated that after 2 hours of hMC stimulation, *S. pneumoniae* was capable of inducing significant amounts of ROS, while cell viability was not affected. However, we cannot exclude that hMC cytotoxicity occurs at later time points.

The unencapsulated *S. pneumoniae* strain D39 (R6) used in the present study serves to understand the dynamics of colonization in mucosal tissue where hMC resides. Previous studies have shown that MC degranulation after encountering encapsulated *S. pneumoniae* only occurs with high bacterial concentrations.^46^, ^47^, ^48^ For instance, Barbuti et al^47^ observed MC degranulation using a MOI of 250:1 with a peak of histamine release after 4 hours. Our study demonstrated that *S. pneumoniae* does not cause either degranulation or chemokine secretion in primary hMCs at a MOI of 25:1. This lack of MC response against *S. pneumoniae* may reflect differences in the capsule status of the strains used, but interestingly this lack of MC response seen in our studies has also been observed in the in vivo infection^49^ where van den Boogaard et al^49^ showed prolonged survival of MC‐deficient mice compared with wild‐type (WT) mice during *S. pneumoniae* infection. Furthermore, the inhibition of MC degranulation in WT mice did not change the disease outcome.^49^ This suggests that during *S. pneumoniae* infection, MCs play a detrimental role for the host, which is independent of degranulation.

Finally, we have investigated hMC responses against *S. aureus*, which is a Gram‐positive coccus colonizing the skin and gut.^50^ The in vivo activity of *S. aureus* on MCs appears diverse and remains controversial and in part may reflect that different *S. aureus* strains have been used in a number of different studies. *S. aureus* has been shown to induce bone marrow–derived murine MCs to release TNF‐α and tryptase.^51^, ^52^ The latter phenomenon also occurs in skin MCs during in vivo infection followed by bacteria internalization.^51^ In a murine peritoneal *S. aureus* infection model, MCs have no impact on the outcome of the disease.^53^ However, in a lung *S. aureus* infection model, MCs display a protective role.^54^ In contrast, our data show a lack of hMC degranulation in response to *S. aureus*, but rather a selective release of a major proinflammatory mediator, PGD2.^55^ Therefore, the in‐vivo mouse data cited above demonstrates that the nature of the tissue influences the type of MC response raised to commensals and pathogens, and our data indicate that hMCs cells exploit differential strategies in antibacterial responses and commensalism compared with their mouse counterparts.

In summary, hMCs control bacterial infections via different mechanisms that are stimulus‐specific and include degranulation, cytokine and chemokine secretion, and ET formation. Our data demonstrated that while *L. monocytogenes* robustly induces degranulation independent of ROS production, *S. pneumoniae* releases ROS with negligible DNA externalization. Furthermore, *E. coli* exhibited a relatively low level of degranulation with higher release of proinflammatory mediators, whereas *S. aureus* selectively released PGD2 without promoting any degranulation. Thus, the present study not only underlines how versatile and plastic hMCs operate in antibacterial immune responses but also how adaptable MCs are in their interaction with commensals.

## CONFLICT OF INTERESTS

The authors declare that there are no conflict of interests.

## AUTHOR CONTRIBUTIONS

KMGR, CS, RB, AG, and SBP participated in the research design. KMGR, CS, and RB conducted the experiments. KMGR, CS, RB, AG, and SBP performed data analysis. ISR contributed to the purification and isolation of bacterial strains. All authors contributed to the writing of the manuscript and reviewed the final version.

## ETHICS STATEMENT

Blood samples were obtained from the NHS blood center of Manchester under the licence REC 2018‐2696‐5711 approved by the University of Manchester Research Ethical Committee (UREC).

## Supporting information

Supporting information

## Data Availability

All the data obtained for this manuscript will be made available by the authors, without undue reservation, to any qualified researcher.
