# Supplementary material for: Human mast cells exhibit an individualized pattern of antimicrobial responses
Source: Immun Inflamm Dis. 2020 Mar 28;8(2):198–210. doi: 10.1002/iid3.295 (PMC7212193; doi:10.1002/iid3.295)
Supplement: Supplementary file 1 — Supporting information [file IID3-8-198-s001.docx]

**Supplemental Figure A**


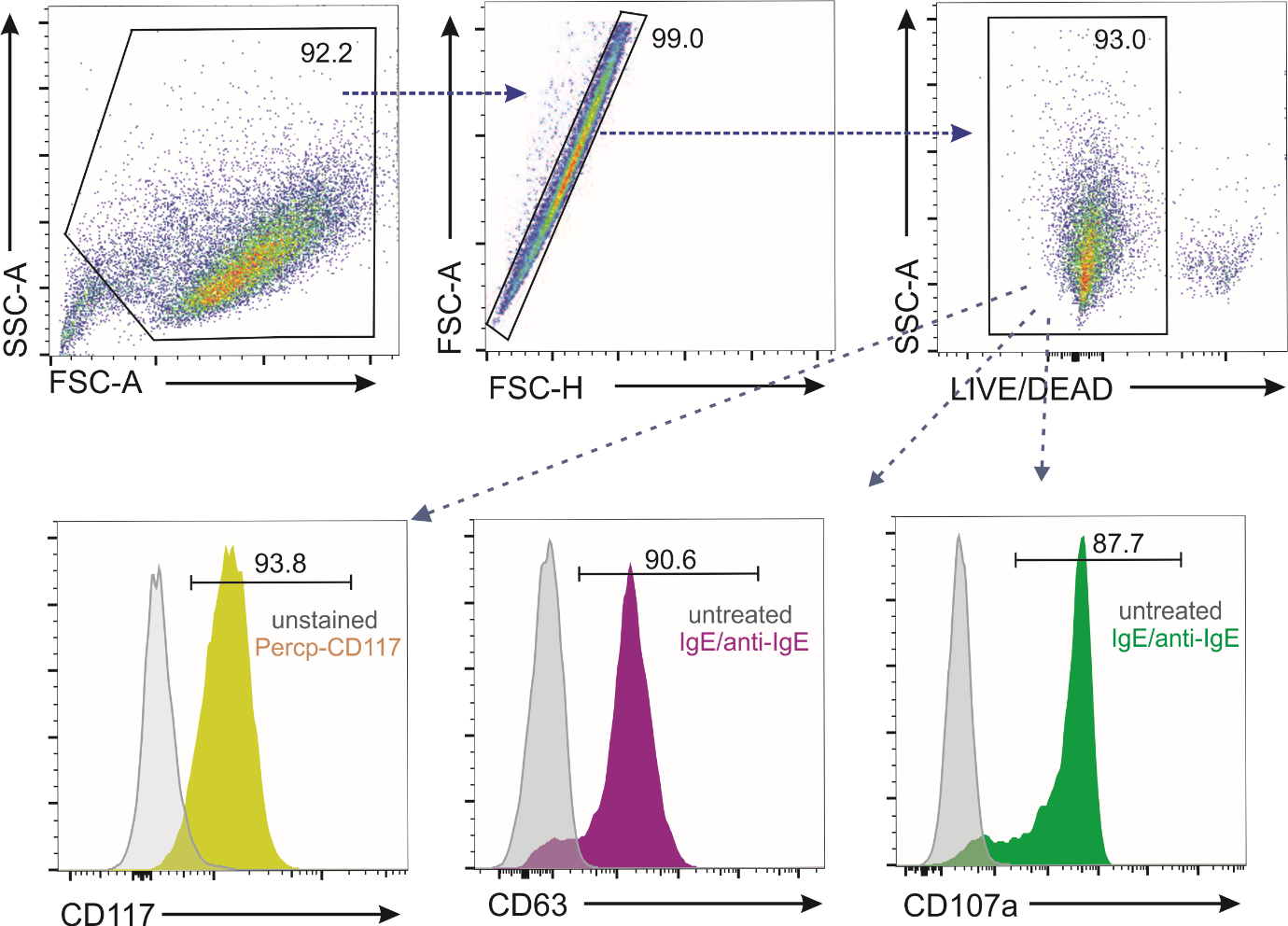


**Supplemental Figure A | Characterization of hMCs.** hMCs isolated from haematopoietic progenitors were cultured for 8 to 10 weeks, and cellular maturity was analysed using flow cytometry. Cells were pre-sensitized with human IgE antibodies, stimulated for 1 hour with anti-IgE antibodies, stained with anti-CD107a, anti-CD63 and anti-CD117 antibodies and DAPI dye and analysed by flow cytometry. (a) Total cell population was selected by side and forward scatter (SSC-A and FSC-A) and single cells were discriminated by forward scatter A and H (FSC-A and FSC-H). Live cells (a) were selected used to gate (b) CD117^+^ cells, (c) CD107a^+^ and CD63^+^ cells (degranulated cells). Fluorescence minus one values (FMO) for CD107a, CD63 and CD117 were used as a control of staining.


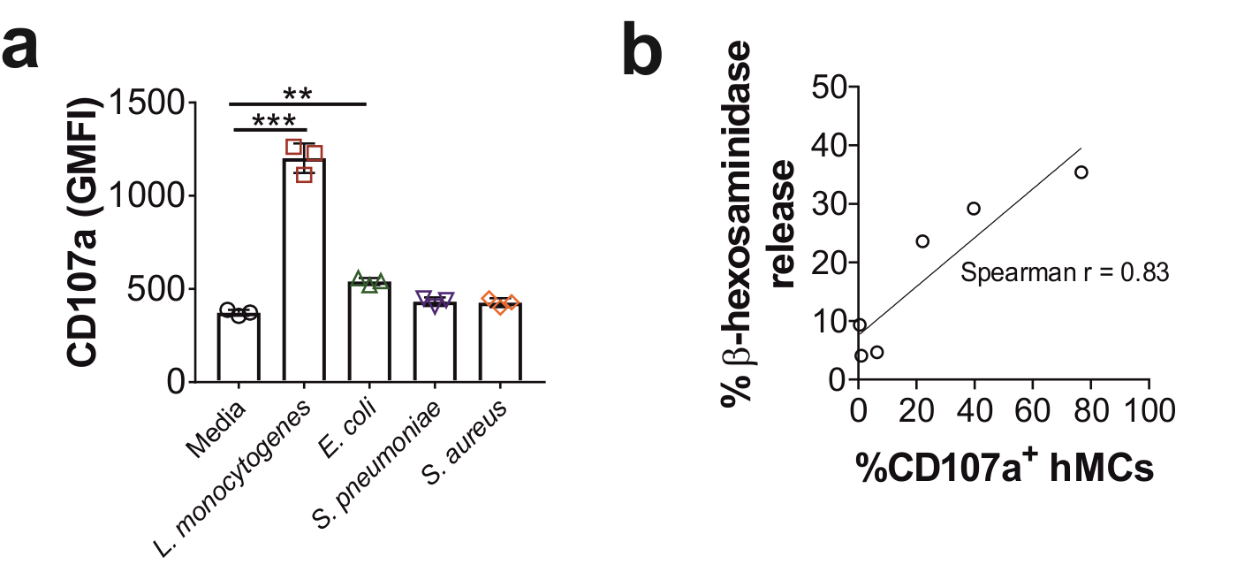
**Supplemental Figure B**

**Supplemental Figure B | Correlation between percentage of β-hexosaminidase release and CD107a expressing cells**

hMCs were stimulated with *L. monocytogenes*, *E. coli*, *S. pneumoniae*, and *S. aureus,* and stained with anti-CD107a antibodies **(a)** bars show the mean of CD107a GMFI of three replicates from one representative experiment out of three independent experiments. Analysis was performed using one-way ANOVA and Tukey’s multiple comparisons post-test (*****p*<0.0001, ****p*<0.001, ***p*<0.01, **p*<0.1). **(b)** hMCs were stimulated with *L. monocytogenes* at a MOI of 25:1 for 2h. β-hexosaminidase release and CD107a expression was measured. The correlation between the two is shown with r_2_ value (Spearman correlation coefficient). Data points show the mean of three independent experiments.

**Supplemental Figure C**


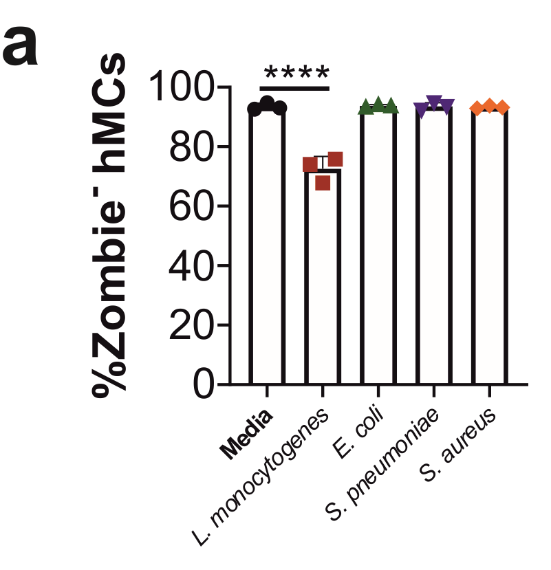


**Supplemental Figure C | Mast cell viability after bacterial stimulation.** Cell viability was studied stimulating hMCs with *L. monocytogenes, E. coli, S. pneumoniae* and *S. aureus* for 2hrs. After stimulation cells were stained with Zombie NIR to evaluate the percentage of live cells. (a) Live cells are represented as Zombie^-^ cells.  Graph shows the mean of three replicates of a representative experiment out of three independent experiments performed. Analysis was calculated using one way ANOVA and Tukey’s multiple comparisons post-test (*****p*<0.0001).
